# An Optimized Real-Time qPCR Method for the Effective Detection of Human Malaria Infections

**DOI:** 10.3390/diagnostics11050736

**Published:** 2021-04-21

**Authors:** Saiful Arefeen Sazed, Mohammad Golam Kibria, Mohammad Shafiul Alam

**Affiliations:** Infectious Diseases Division, International Centre for Diarrheal Diseases Research, Bangladesh Mohakhali, Dhaka 1212, Bangladesh; saiful.sazed@icddrb.org (S.A.S.); golam.kibria@icddrb.org (M.G.K.)

**Keywords:** *Plasmodium*, malaria, real time PCR, asymptomatic, diagnosis, nested PCR

## Abstract

Polymerase chain reaction, although an expensive method for the detection of human *Plasmodium* spp., is still considered the finest for the diagnosis of malaria. The conventional diagnostic PCR is an inexpensive process but consumes a lot of time, reagents and lacks sensitivity. On the other hand, real-time PCR assays currently being used are mostly probe-based expensive methods and sometimes not feasible to detect all the species in a single amplification reaction condition. Here we have established a real-time PCR method that is time and cost effective with a single protocol to detect and distinguish five human *Plasmodium* species using the existing primers efficiently. The primers used here are being used in the conventional method and the sensitivity as well as specificity of this method has also been immensely improved (100%). The lower limit of detection for *Plasmodium falciparum*, *Plasmodium vivax* and *Plasmodium malariae* are 0.064 parasites/µL, 1.6 parasites/µL, and 0.32 parasites/µL respectively and no cross reactivity was observed. Besides, we have analyzed melt curves that can be used for further species confirmation and validation purposes using multiplex systems. This method, therefore, can be considered as an alternative to the existing lineup for molecular diagnosis of malaria in endemic countries.

## 1. Introduction

Malaria is a deadly mosquito-borne parasitic disease with an estimated 409,000 deaths and 229 million cases in 2019 [1], most of which are children on the continent of Africa [2]. Besides, more than thousands of cases are also observed in non-endemic countries like the United States, mostly due to travelers returning from endemic countries [3,4]. Many endemic countries are now heading towards the elimination of malaria [5] and have taken initiatives to point out the burdens and solutions to those hindrances one of which is asymptomatic cases [6]. Although asymptomatic cases cannot be easily defined by the simple parasite density nonetheless it is evident that there is a clear relationship between low parasitemiae and asymptomatic burden [7]. In most cases, the previous studies suggest that these asymptomatic populations are easily filtered out during diagnosis when the methods are RDT, microscopy, and/or even conventional PCR [8,9]. Even though some modern technologies such as rotating-crystal magneto-optical detection (RMOD), magnetic resonance relaxometry (MRR), and surface enhanced Raman spectroscopy (SERS) have high sensitivity, they are still on the developmental stage to meet the feasibility at point of care detection [10,11,12,13]. Therefore, proper diagnosis is a vital element for choosing an appropriate treatment regimen based on the nature of the infection.

Besides, severe malaria should also be taken into consideration as it is responsible for causing death. Severe malaria, predominantly caused by *Plasmodium falciparum* (*Pf*), can be portrayed as a multi-system disorder with a wide range of clinical features including destruction of red blood cells (RBC) and stroke due to adhesion and sequestration of parasites resulting in anemia and cerebral malaria [14]. Recent evidences show us the possibility of severe malaria by *Plasmodium vivax* (*Pv*) as well which is alarming if not monitored closely as it can render severe Acute Respiratory Distress Syndrome (ARDS) [15,16]. Thus a mass sero-surveillance with a sensitive diagnostic method is required to reduce such toll of deaths by malaria.

Traditionally in the endemic regions, the primary method of initial parasite detection is Giemsa-stain based microscopy [17] and it may require expertise to differentiate among the species [18,19]. The immuno-chromatographic approaches in the form of the rapid diagnostic test (RDT) are also popular that are very rapid but less sensitive in the case of low parasitemia (<100–200 parasites/µL) [20,21] and often, is not an indicator of current infection that may render a false diagnosis of active infection [22]. In many endemic countries, conventional gel electrophoresis based PCR is mostly used for diagnostic purposes at the central level [23]. But the process itself is very time consuming, requires a bulk amount of reagents and multiple steps in the processes, and lacks sensitivity (sensitivity > 10 parasites/µL) [24,25].

The aforementioned problems can be easily resolved using modern technology such as real-time qPCR, digital PCR (dPCR), and digital droplet PCR (ddPCR). These state-of-the-art methods have the capacity to detect even low parasite density (<0.1 parasites/µL) [26,27,28,29]. The qPCR method is still sensitive and efficient enough to detect these cases. But most of the sensitive qPCR methods, currently being used, employs probe-based approaches that are expensive and often cannot be used in single reaction conditions (different annealing, elongation temperature, and/or cycle numbers, etc.) [30]. In this study, we aimed to develop a new time, cost, and process efficient approach using the existing primers for the detection and distinguishing all five human malaria-causing parasites i.e., *P. falciparum*, *P. vivax*, *P. malariae* (*Pm*)*, P. ovale* (*Po*), and *P. knowlesi* (*Pk*) in a single amplification reaction condition using extracted genomic DNA. We have also proposed using multiplex systems with melt curve analysis data considering the nature of malaria infection in individual endemic countries.

## 2. Materials and Methods

### 2.1. Specimens

Plasmid controls of different *Plasmodium* species collected from the American Type Culture Collection (Manassas, VA, USA) and culture controls were employed for the method development process initially (*Pf*-*MRA*-177, *Pv*-MRA-178, *Pm*-MRA-179, *Po*-MRA-180, and *Pk* culture control from the University of Malaya, Kuala Lumpur, Malaysia). Archived DNAs of clinical positive samples were also used as endemic control to measure the clinical sensitivity and specificity. All the samples were collected for previous studies from several malaria-endemic districts of Bangladesh (Khagrachari, Cox’s Bazar, Rangamati, and Netrokona) [31,32]. The original studies were approved by the Ethics Review Committee of the International Centre for Diarrhoeal Disease Research, Bangladesh. Informed consent was taken from every participant for future use of the sample. DNA was extracted from whole blood using the QIAamp Blood Mini Kit (Qiagen, Hilden, Germany).

### 2.2. Real-Time PCR

CFX-96 real-time PCR detection system (Bio-Rad, Hercules, CA, USA) was employed for the study. The primers used here had been previously described in a conventional PCR system (Supplementary S1: Table S1) [24,25,33,34]. For each reaction 0.7 µL 10 uM forward and reverse primers, 10 µL 2× iQ SYBR green supermix (Bio-Rad), and 5.6 µL nuclease-free water comprising 17 µL of Mastermix was used and a DNA sample of 3 µL was taken from each extracted DNA sample. The PCR protocol includes an initial denaturation at 95 °C for 10 min, followed by amplification for a 35 cycle of 1 min denaturation at 95 °C, 15-second annealing at 57 °C, and 30-s elongation step at 61 °C. The fluorescence absorption values were acquired at the end of every elongation step. The steps were optimized through gradient run as well as trial and error. Separate sets of primers were employed for each reaction for the detection of each species. The whole experiment took only about two hours in total including an additional melt-curve analysis. For the melt curve analysis, an additional melt program was employed with an initial 60 °C for 10 min followed by a stepwise increase of 0.5 °C from 65 °C until 85 °C.

### 2.3. Multiplex System

Multiplex assays were also performed using two to three sets of primers for detecting species using melting curve analysis. This additional system employed of *Pf*, *Pv*, and *Pm*; *Pf* and *Pv*; *Pv* and *Pm* primers combination system in individual wells were also employed with single positive template and co-infected sample templates as well. This technique was applied to determine the possibility of species detection using melting curve analysis as a single species always has a distinct melting temperature (T_m_).

### 2.4. Analytical Sensitivity, Specificity, Linear and Repeatability

For analytical sensitivity and linearity, parasites cultivated from the *P. falciparum* 3d7 strain were collected and diluted with fresh RBC to obtain parasitemia from 5000 parasites/µL to as low as 0.002 parasites/µL (diluted five times in each step) whereas, for *P. vivax* and *P. malariae*, archived patient samples with 25,000 parasites/µL and 1000 parasites/µL were diluted to obtain 1.6 parasites/µL and 0.32 parasites/µL respectively (also 5 times diluted in each step). The samples were properly mixed to ensure homogeneity and genomic DNA extraction was performed on the serially diluted samples individually using QIAGEN DNA BLOOD Mini kit (Qiagen) following the manufacturer’s instruction (Supplementary S1: Section S2). Intra-assay and inter-assay repeatability were checked in triplicate reaction. Variability was analyzed in terms of coefficient of variation (CV; the ratio of mean to the standard deviation (SD)) among intra and inter-assay ct values in triplicates. For intra assay, triplicate values and for inter assay, average of individual triplicate ct values were calculated.

For specificity, individual primers were employed with plasmid control templates of other species in separate amplification reactions. Moreover, positive template controls of other pathogens including trypanosome parasites *Leishmania* spp., apicomplexan parasite *Cryptosporidium* spp., *Giardia* spp., *Strongyloides* spp., gram-negative bacteria *Escherichia coli*, *Shigella*, *Campylobacter*, *Vibrio cholera*, Adenovirus, *Norovirus*, *Rotavirus*, etc. have also been used to rule out cross-reactivity.

### 2.5. Clinical Sensitivity and Specificity

The clinical sensitivity and specificity of this PCR assay method for the detection of *Pf*, *Pv*, and *Pm* were calculated considering the microscopic examination as the gold standard in the initial diagnosis for malaria infection. For that purpose, we have performed the assay on 50 *Pf*, 40 *Pv*, and 20 *Pm* positive clinical samples. An equivalent amount of endemic *Plasmodium* sp. negative samples were also taken for analysis of specificity and sensitivity of the individual species. Sensitivity and specificity were calculated using binomial methods for proportion with a 95% confidence interval (CI) [35].

### 2.6. Reagent Cost Analysis

The associated reagent costs were estimated compared to conventional and probe- based qPCR. As DNA extraction is equal for all the PCR methods, cost of extraction kit was not considered. Costs of research infra-structure and personnel were excluded. For estimation of cost, a batch size of 30 samples were estimated [36,37].

## 3. Results

### 3.1. Detection and Melting Curve Analysis

**Figure 1:**
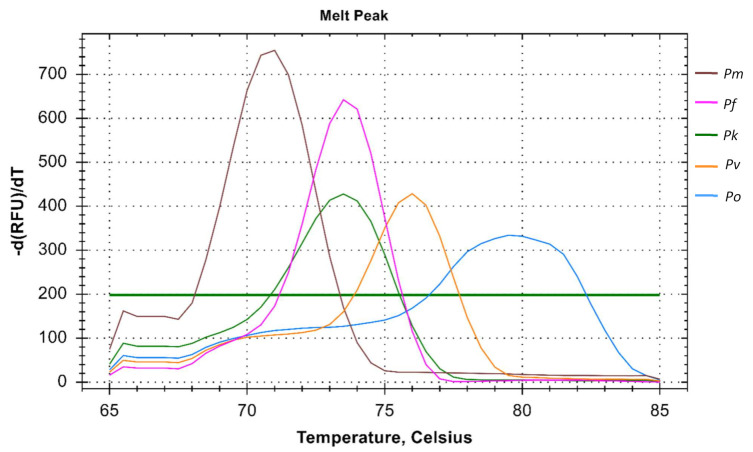
Melt curve analysis of *Plasmodium* spp. The melt curve shows different T_m_ for *Pf* (73.5 °C), *Pv* (76 °C), *Pm* (71 °C), and *Po* (79.5 °C) except for *Pk* (73.5 °C) which overlaps with the *Pf*.

**Figure 2:**
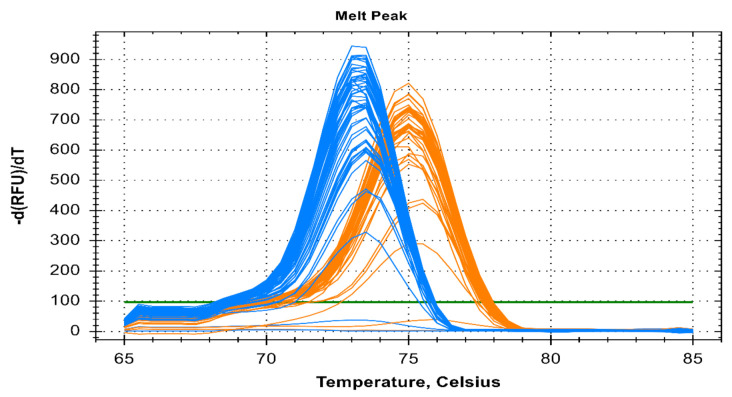
Melt curve analysis of clinical fifty *Pf* and forty *Pv* samples depicted by blue and orange color respectively. The melting temp (T_m_) for *Pf* and *Pv* of clinical samples were 73.5 °C ± 0.5 °C and 75 °C ± 0.5 °C respectively.

**Figure 3:**
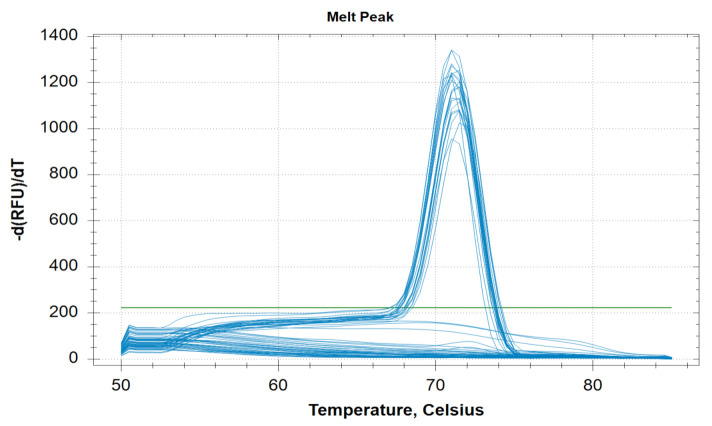
Melting curve analysis of clinical twenty clinical *Pm* samples with melting temperature (T_m_) of 71 °C ± 0.5 °C.

This PCR method can detect five different species of human malaria parasite effectively under the same reaction parameter. The melt curve analysis also shows us the different T_m_ for all the different species except for *Pk* that overlaps with the *Pf* at 73.5 °C. The T_m_ for *Pv*, *Pm*, and *Po* are 76 °C, 71 °C, and 79.5 °C respectively for plasmid controls (Figure 1). In clinical samples, the T_m_ for *Pf*, *Pv*, and *Pm* were found almost identical (73.5 ± 0.5 °C, 75.0 ± 0.5 °C, and 71 ± 0.5 °C) (Figure 2 and Figure 3).

**Figure 4:**
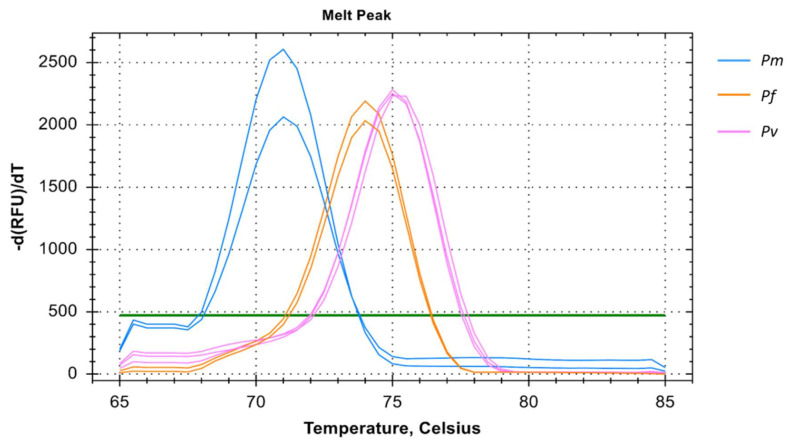
Melting curve analysis of *Pm*, *Pf*, and *Pv* samples in multiplex depicted by blue, orange, and pink colors respectively. The melting temperature (T_m_) for *Pm*, *Pf*, and *Pv* of samples were 71 °C, 74 °C, and 75 °C respectively. The multiplex melting curve analysis could only verify mono-infection with distinct melting temperatures for each species.

It is evident that individual mono-infection can be determined using the multiplex primer systems i.e., primers for *Pf*, *Pv*, and *Pm* in a single amplification reaction with T_m_ of 74 °C, 75 °C, and 71 °C respectively indicating mono-infection only (Figure 4). For every mono-infection, a distinct melting temperature was observed but co-infection could not be determined in all the run. This was consistent in both two and three primer multiplex system.

### 3.2. Analytical Sensitivity, Linearity, and Reproducibility of the Assay

**Figure 5:**
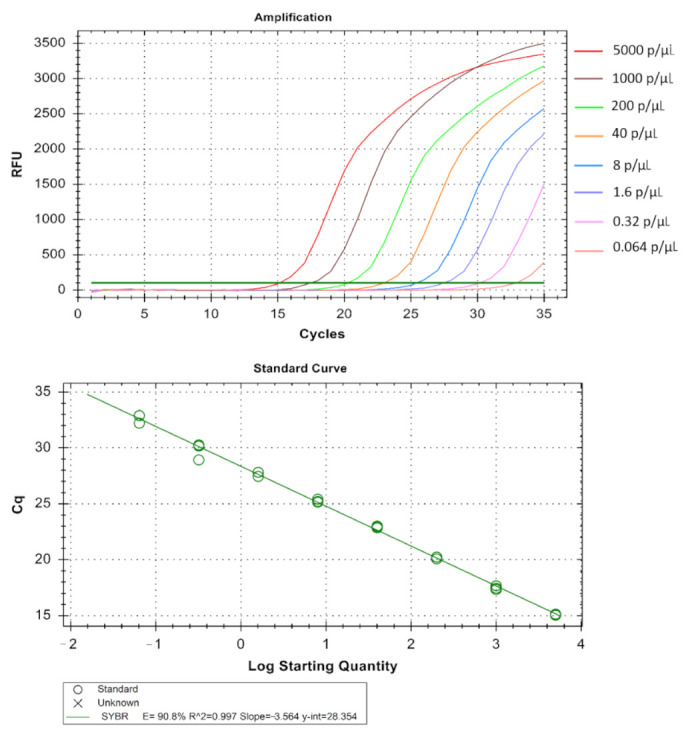
Technical performance and range of detection of the *Pf* real-time SYBR green PCR. DNA was extracted from MRA-102 3D7 strain cultured in vitro, ranging from 5 × 10^3^ to 6.4 × 10^−2^ parasites/µL, and subjected to real-time SYBR green based PCR. Amplification curves are depicted by different colors each containing a different parasite load. The standard curve has been made by the ct values obtained from the assay plotted against the logarithmic parasite concentration in a linear regression curve fit analysis (R^2^ = 0.997, E = 90.8%).

**Figure 6:**
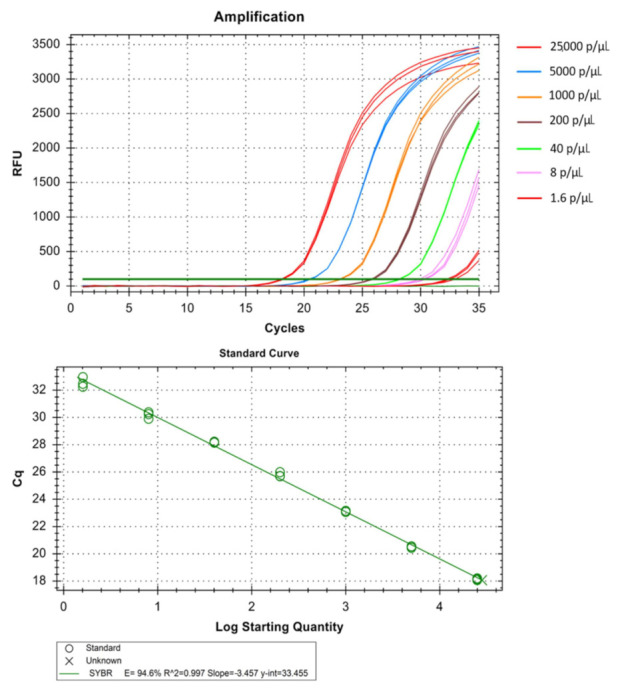
Technical performance and range of detection of the *Pv* real-time SYBR green PCR. Amplification curves of archived *Pv* samples with concentration ranging from 25 × 10^3^ to 1.6 parasites/µL, have been depicted by different colors each containing a different parasite load. The standard curve has been made by the ct values obtained from the assay plotted against the logarithmic parasite concentration in a linear regression curve fit analysis (R^2^ = 0.997, E = 94.6%).

**Figure 7:**
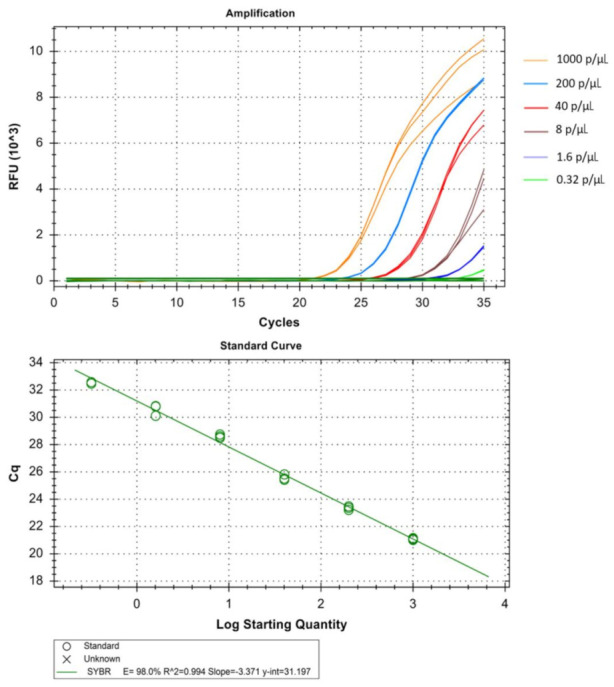
Technical performance and range of detection of the *Pm*, real-time SYBR green PCR. Amplification curves of archived *Pm* samples with concentration ranging from 1 × 10^3^ to 3.2 × 10^−1^ parasites/µL, have been depicted by different colors each containing a different parasite load. The standard curve has been made by the ct values obtained from the assay plotted against the logarithmic parasite concentration in a linear regression curve fit analysis (R^2^ = 0.994, E = 98.0%).

The real-time assay can detect as low as 0.064 parasites/µL in *Pf* (Table 1 and Figure 5), 1.6 parasites/µL in *Pv* (Table 2 and Figure 6) and 1.6 parasites/µL in *Pm* (sometimes as low as 0.32 parasites/ µL) (Table 3 and Figure 7). The standard curve had been generated with 5 times dilution in each step and over eight, seven, and six concentration range in *Pf*, *Pv*, and *Pm* respectively. The correlation coefficients (R^2^) were ≥0.994 and efficiency over 90% in all cases (Figure 5, Figure 6 and Figure 7). The amplification was further verified through gel electrophoresis analysis of the product (Supplementary S2: Figures S1–S3). The amplification reaction did not show any appearance of ct value in the non-template control that indicates an absence of any nonspecific amplification and/or contamination. Therefore, the assay was highly specific for all the individual *Plasmodium* spp. The intra-assay CV of ct values for *Pf* ranged from 0.34% to 2.58% over eight different concentrations and for *Pv* it was 0.13% to 1.11% over the seven concentrations range. For *Pm*, the intra assay CV of ct values ranged from 0.31% to 1.31% over six different concentration levels. The increased variations were observed in low parasite load only. Reproducibility of the assay was assessed through inter-assay variation of ct values for the same concentration level in two different independent runs. The CV for inter-assay fluctuated from 0.45% to 2.11%, 0.30% to 1.58% and 0.24% to 1.20% for *Pf*, *Pv* and *Pm* respectively. The CV, therefore, indicates high reproducibility of the assay method.

### 3.3. Clinical Specificity and Cross-Reactivity

The clinical sensitivity and specificity were 100% for *Pf* (sensitivity 95% CI: 91.11– 100%; specificity 95% CI: 91.11–100%), *Pv* (sensitivity 95% CI: 89.09–100%; specificity 95% CI: 91.11–100%) and *Pm* (sensitivity 95% CI: 79.95–100%; specificity 95% CI: 89.09–100%). The PCR assay was highly specific in respect to other species of the same genus and showed no cross-reactivity for the aforementioned species of other genera with the absence of a peak in cross-reactive amplification reactions as well.

### 3.4. Comparison of Cost-Effectiveness

The cost of per reaction was highest in the conventional gel-electrophoresis based PCR approach with 3.7 US$ per sample. The probe based qPCR approach costs around 1.8 US$ while this SYBR green based approach requires the lowest with 0.75 US$ per sample. The costs were estimated using the standard average price of the commercial supply kits. The detailed comparison can be observed in the Table 4.

## 4. Discussion

This qPCR method requires 35 cycles but amplification was also performed for 40 cycles. It was evident that this extended number of cycles increases the sensitivity of the reaction but sometimes that hampers the strand specificity which is a common limitation of the Sybr green-based PCR. Besides, almost similar results were obtained with 58 °C and 60 °C for annealing and elongation steps respectively. For analysis of data, the lower cut-off ct value was set as 33 to consider the sample as positive with a baseline threshold of 100. Increasing the time in between amplification steps in the annealing and elongation stage also showed good results but it increases the experimental time-length.

In terms of sensitivity, the conventional gel electrophoresis based PCR could detect 6–31 parasites/µL when nested PCR was performed and it drastically reduces when oligonucleotides are used in single amplification reaction as opposed to nested PCR (<3000 parasites/µL) [34]. In a previous probe based study using reverse transcriptase qPCR employed extraction of DNA and RNA from small volume of dried blood in RDTs could detect 1 parasites/µL in asymptomatic patients [38]. Another study employed the *Pf* HRP II/III (Histidine rich protein) deletions with lower limit of detection (LOD) 3 parasites/µL [39] whereas in a diagnostic probe based study conducted on 297 patients could detect four species except *Po* and fails to evaluate mixed infections as well with sensitivity of 95% only [40]. The major problem is often observed for detection of simian malaria *Pk* that also causes infection in human. Besides, the commercial kit available to detect *Pk* often shows false positive result in mixed infection at low parasitemia with a LOD of 0.125 and 20 parasites/µL only [41]. Targeting high copy telomere associated repetitive element and var gene acidic terminal achieved a LOD of 0.03 to 0.15 parasites/µL for detection of *Pf* [42] while 18s rRNA target based approach in clinical isolates resulted in detection of *Pf* and *Pv* wih LOD of 1–10 parasites/µL with 100% sensitivity [43]. In our previous SYBR green based study, the lower limit of detection was 5–10 parasites/µL for *Pf* and *Pv* with sensitivity and specificity below 100% [31].

In contrast, this method can detect as low as 0.64 to 1.6 parasites/µL in *Pf*, *Pv*, and *Pm* and thus dictates the high sensitivity of the method. Analytical sensitivity, linearity and repeatability could not be performed on the *Po* and *Pk* as there are no clinical isolates in our research facilities. The method can also utilize multiplex system efficiently in contrast to the conventional method that failed to detect specific species with a marked decrease in sensitivity. Another mentionable benefit of the method is that five of the human malaria species can be run under the same reaction condition whereas in nested conventional PCR we can perform only four species. Besides in nested PCR, we have to run the PCR for amplification of the common genus region gene first and then go for the species amplification which is a time consuming process [34]. In case of qPCR, most of the protocols currently being used employ different temperature conditions for different *Plasmodium* species or different sets of species [44,45,46,47]. Thus, this process has greatly improved the detection process through a significant reduction of time and steps.

This multiplex system can be deployed for diagnostic purposes in malaria-endemic regions. For that purpose, we can detect initially the most common causative endemic species such as *Pf* and/or *Pv* and later use the multiplex system to determine the other species. However, the multiplex of *Pf*, *Pv*, and *Pm* can also be used in a single reaction but that can detect mono-infection only. The reason behind the lacking in the detection of co-infection can be explained by the common flaw of SYBR green-based chemistry in the PCR system [48].

## 5. Conclusions

This method as described is time and cost efficient as it takes less than a US$ for each PCR reaction and about two US$ if the multiplex system is used. Compared to probe based and conventional methods it is 2.5-fold and about 5-fold cheaper respectively (Supplementary S1). Low intra and inter assay CV of this method also is also an indicator of the high reproducibility of the PCR assay. The steps have been reduced greatly here compared to the existing conventional gel electrophoresis based method usually used for the diagnostic approach. On the other hand, the Taqman probe-based approach can be more efficient but increases the cost of each reaction and thus not suitable for cost-effective diagnostic purposes. In terms of time, cost, steps, and expertise, we recommend that this method can be an excellent convenient alternative approach and be added to the existing current lineup of diagnostic tools.

## Figures and Tables

**Table 1:** Reproducibility and repeatability of real-time PCR assay for *Pf*.

	Intra Assay Variation of CT Values						Inter Assay Variation of CT Values				
Parasites/µL	Replicate 1	Replicate 2	Replicate 3	Mean	SD	CV	Assay 1	Assay 2	Mean	SD	CV
5000	15.14	15.11	15.04	15.10	0.05	0.34	15.10	15.23	15.16	0.10	0.63
1000	17.46	17.67	17.34	17.49	0.17	0.96	17.49	17.68	17.59	0.14	0.78
200	20.25	20.23	20.08	20.19	0.10	0.47	20.19	20.32	20.25	0.09	0.45
40	23.02	22.85	22.95	22.94	0.08	0.35	22.94	22.66	22.80	0.20	0.86
8	25.41	25.21	25.15	25.26	0.14	0.55	25.26	26.01	25.63	0.53	2.08
1.6	27.44	27.82	27.91	27.72	0.25	0.89	27.72	28.08	27.90	0.25	0.89
0.32	30.20	30.30	28.92	29.81	0.77	2.58	29.81	30.71	30.26	0.64	2.11
0.064	32.94	32.88	32.27	32.70	0.38	1.15	32.70	32.47	32.58	0.16	0.50

**Table 2:** Reproducibility and repeatability of real-time PCR assay for *Pv*.

	Intra Assay Variation of CT Values						Inter Assay Variation of CT Values				
Parasites/µL	Replicate 1	Replicate 2	Replicate 3	Mean	SD	CV	Assay 1	Assay 2	Mean	SD	CV
25,000	18.15	18.20	18.07	18.14	0.06	0.36	18.14	18.25	18.20	0.08	0.42
5000	20.55	20.53	20.44	20.51	0.06	0.28	20.51	20.59	20.55	0.06	0.30
1000	23.15	23.07	23.15	23.12	0.05	0.21	23.12	23.28	23.20	0.11	0.48
200	25.69	25.71	25.99	25.79	0.17	0.65	25.79	25.75	25.77	0.03	0.12
40	28.19	28.22	28.15	28.19	0.04	0.13	28.19	28.23	28.21	0.03	0.11
8	30.24	29.91	30.38	30.18	0.24	0.81	30.18	30.86	30.52	0.48	1.58
1.6	32.96	32.26	32.47	32.56	0.36	1.11	32.56	32.74	32.65	0.12	0.38

**Table 3:** Reproducibility and repeatability of real-time PCR assay for *Pm*.

	Intra Assay Variation of CT Values						Inter Assay Variation of CT Values				
Parasites/µL	Replicate 1	Replicate 2	Replicate 3	Mean	SD	CV	Assay 1	Assay 2	Mean	SD	CV
1000	21.08	21.02	21.15	21.08	0.07	0.31	21.08	21.26	21.17	0.13	0.59
200	23.22	23.37	23.48	23.35	0.13	0.56	23.35	23.43	23.39	0.06	0.24
40	25.43	25.52	25.82	25.59	0.20	0.80	25.59	25.96	25.77	0.26	1.01
8	28.60	28.74	28.50	28.61	0.12	0.42	28.61	28.35	28.48	0.19	0.67
1.6	30.11	30.79	30.82	30.57	0.40	1.31	30.57	30.93	30.75	0.25	0.82
0.32	32.58	--	32.47	32.52	0.07	0.23	32.52	33.08	32.80	0.39	1.20

**Table 4:** Cost distribution and comparison among conventional nested, Sybr green (this method) and Taqman probe-based PCR approaches.

Conventional Nested PCR (Batch of 30 Samples for a Single Parasite)	
Components	Overall Cost per Sample (US $)
Buffer	0.2
dNTPs	0.7
Primers (4 Sets)	0.4
Taq Polymerase	0.3
Nuclease-Free Water	0.1
Agarose	0.4
TBE Buffer	0.3
Gel-Red	0.2
Gel Loading Dye	0.1
DNA Ladder	0.2
Consumables (PCR Tube, Gel Loading Plate, Tips, Distilled Water etc.)	0.8
Overall Cost per Sample (USD)	3.7
**SYBR Green qPCR (Batch of 30 Samples for a Single Parasite)**	
**Components**	**Overall Cost per Sample (US $)**
SYBR Green PCR Supermix	0.15
Primers (2 Sets)	0.2
Nuclease-Free Water	0.1
Consumables (PCR plate/Tube, Tips etc.)	0.3
Overall Cost per Sample (USD)	0.75
**Taqman qPCR (Batch of 30 Samples for a Single Parasite)**	
**Components**	**Overall Cost per Sample (US $)**
Taqman qPCR Supermix	0.25
Primers (2 sets)	0.2
Probe (1 set)	0.95
Nuclease-Free Water	0.1
Consumables (PCR plate/Tube, Tips etc.)	0.3
Overall Cost per Sample (USD)	1.8

## Data Availability

Not applicable.

## Supplementary Materials

The following are available online at https://www.mdpi.com/article/10.3390/diagnostics11050736/s1, two supplementary files have been added with this submission. Supplementary S1 with Table S1 and Qiagen DNA Kit insertions. Supplementary S2 contains 3 figures of gel electrophoresis confirmation.

## References

[1] World Health Organization (2020). World Malaria Report 2020.

[2] Schumacher R.-F., Spinelli E. (2012). Malaria in children. Mediterr. J. Hematol. Infect. Dis..

[3] Crockett M.E., Keystone J.S. (2008). Protection of Travelers. Principles and Practice of Pediatric Infectious Diseases.

[4] Angelo K.M., Libman M., Caumes E., Hamer D.H., Kain K.C., Leder K., Grobusch M.P., Hagmann S.H., Kozarsky P., Lalloo D.G. (2017). Malaria after international travel: A GeoSentinel analysis, 2003–2016. Malar. J..

[5] Dhiman S. (2019). Are malaria elimination efforts on right track? An analysis of gains achieved and challenges ahead. Infect. Dis. Poverty.

[6] Adhikari B., Phommasone K., Pongvongsa T., Soundala X., Koummarasy P., Henriques G., Peto T.J., von Seidlein L., White N.J., Day N.P.J. (2018). Perceptions of asymptomatic malaria infection and their implications for malaria control and elimination in Laos. PLoS ONE.

[7] Lindblade K.A., Steinhardt L., Samuels A., Kachur S.P., Slutsker L. (2013). The silent threat: Asymptomatic parasitemia and malaria transmission. Expert Rev. Anti-Infect. Ther..

[8] Rodríguez Vásquez C., Escobar S.B., Tobón-Castaño A. (2018). Low Frequency of Asymptomatic and Submicroscopic Plasmodial Infections in Urabá Region in Colombia. J. Trop. Med..

[9] Bousema T., Okell L., Felger I., Drakeley C. (2014). Asymptomatic malaria infections: Detectability, transmissibility and public health relevance. Nat. Rev. Microbiol..

[10] Arndt L., Koleala T., Orbán Á., Ibam C., Lufele E., Timinao L., Lorry L., Butykai Á., Kaman P., Molnár A.P. (2021). Magneto-optical diagnosis of symptomatic malaria in Papua New Guinea. Nat. Commun..

[11] Peng W.K., Kong T.F., Ng C.S., Chen L., Huang Y., Bhagat A.A.S., Nguyen N.T., Preiser P.R., Han J. (2014). Micromagnetic resonance relaxometry for rapid label-free malaria diagnosis. Nat. Med..

[12] Veiga M.I., Peng W.K. (2020). Rapid phenotyping towards personalized malaria medicine. Malar. J..

[13] Chen K., Yuen C., Aniweh Y., Preiser P., Liu Q. (2016). Towards ultrasensitive malaria diagnosis using surface enhanced Raman spectroscopy. Sci. Rep..

[14] Mackintosh C.L., Beeson J.G., Marsh K. (2004). Clinical features and pathogenesis of severe malaria. Trends Parasitol..

[15] Gupta H., Afsal M.P., Shetty S.M., Satyamoorthy K., Umakanth S. (2015). *Plasmodium vivax* infection causes acute respiratory distress syndrome: A case report. J. Infect. Dev. Ctries..

[16] Anvikar A.R., van Eijk A.M., Shah A., Upadhyay K.J., Sullivan S.A., Patel A.J., Joshi J.M., Tyagi S., Singh R., Carlton J.M. (2020). Clinical and epidemiological characterization of severe *Plasmodium vivax* malaria in Gujarat, India. Virulence.

[17] Ugah I.U., Alo M.N., Owolabi J.O., Okata-Nwali O.D., Ekejindu I.M., Ibeh N., Elom M.O. (2017). Evaluation of the utility value of three diagnostic methods in the detection of malaria parasites in endemic area. Malar. J..

[18] Ortiz Ruiz A., Postigo M., Casanova S.G., Cuadrado D., Bautista J.M., Rubio J.M., Luengo-Oroz M., Linares M. (2018). *Plasmodium* species differentiation by non-expert on-line volunteers for remote malaria field diagnosis. Malar. J..

[19] Azikiwe C.C.A., Ifezulike C.C., Siminialayi I.M., Amazu L.U., Enye J.C., Nwakwunite O.E. (2012). A comparative laboratory diagnosis of malaria: Microscopy versus rapid diagnostic test kits. Asian Pac. J. Trop. Biomed..

[20] Berhane A., Russom M., Bahta I., Hagos F., Ghirmai M., Uqubay S. (2017). Rapid diagnostic tests failing to detect *Plasmodium falciparum* infections in Eritrea: An investigation of reported false negative RDT results. Malar. J..

[21] Mwesigwa J., Slater H., Bradley J., Saidy B., Ceesay F., Whittaker C., Kandeh B., Nkwakamna D., Drakeley C., van Geertruyden J.P. (2019). Field performance of the malaria highly sensitive rapid diagnostic test in a setting of varying malaria transmission. Malar. J..

[22] Dalrymple U., Arambepola R., Gething P.W., Cameron E. (2018). How long do rapid diagnostic tests remain positive after anti-malarial treatment?. Malar. J..

[23] Johnston S.P., Pieniazek N.J., Xayavong M.V., Slemenda S.B., Wilkins P.P., da Silva A.J. (2006). PCR as a confirmatory technique for laboratory diagnosis of malaria. J. Clin. Microbiol..

[24] Snounou G., Viriyakosol S., Zhu X.P., Jarra W., Pinheiro L., Rosario V.E.d., Thaithong S., Brown K.N. (1993). High sensitivity of detection of human malaria parasites by the use of nested polymerase chain reaction. Mol. Biochem. Parasitol..

[25] Snounou G., Singh B., Doolan D.L. (2002). Nested PCR Analysis of *Plasmodium* Parasites. Malaria Methods and Protocols: Methods and Protocols.

[26] Taylor S.M., Juliano J.J., Trottman P.A., Griffin J.B., Landis S.H., Kitsa P., Tshefu A.K., Meshnick S.R. (2010). High-throughput pooling and real-time PCR-based strategy for malaria detection. J. Clin. Microbiol..

[27] Perandin F., Manca N., Calderaro A., Piccolo G., Galati L., Ricci L., Medici M.C., Arcangeletti M.C., Snounou G., Dettori G. (2004). Development of a real-time PCR assay for detection of *Plasmodium falciparum*, *Plasmodium vivax*, and *Plasmodium ovale* for routine clinical diagnosis. J. Clin. Microbiol..

[28] Rougemont M., van Saanen M., Sahli R., Hinrikson H.P., Bille J., Jaton K. (2004). Detection of four *Plasmodium* species in blood from humans by 18S rRNA gene subunit-based and species-specific real-time PCR assays. J. Clin. Microbiol..

[29] Veron V., Simon S., Carme B. (2009). Multiplex real-time PCR detection of *P. falciparum*, *P. vivax* and *P. malariae* in human blood samples. Exp. Parasitol..

[30] Haanshuus C.G., Mørch K., Blomberg B., Strøm G.E.A., Langeland N., Hanevik K., Mohn S.C. (2019). Assessment of malaria real-time PCR methods and application with focus on low-level parasitaemia. PLoS ONE.

[31] Alam M.S., Mohon A.N., Mustafa S., Khan W.A., Islam N., Karim M.J., Khanum H., Sullivan D.J.S., Haque R. (2011). Real-time PCR assay and rapid diagnostic tests for the diagnosis of clinically suspected malaria patients in Bangladesh. Malar. J..

[32] Mohon A.N., Alam M.S., Bayih A.G., Folefoc A., Shahinas D., Haque R., Pillai D.R. (2014). Mutations in *Plasmodium falciparum* K13 propeller gene from Bangladesh (2009–2013). Malar. J..

[33] Singh B., Sung L.K., Matusop A., Radhakrishnan A., Shamsul S.S.G., Cox-Singh J., Thomas A., Conway D.J. (2004). A large focus of naturally acquired *Plasmodium knowlesi* infections in human beings. Lancet.

[34] Singh B., Bobogare A., Cox-Singh J., Snounou G., Abdullah M.S., Rahman H.A. (1999). A genus- and species-specific nested polymerase chain reaction malaria detection assay for epidemiologic studies. Am. J. Trop. Med. Hyg..

[35] Hossain F., Ghosh P., Khan M.A.A., Duthie M.S., Vallur A.C., Picone A., Howard R.F., Reed S.G., Mondal D. (2017). Real-time PCR in detection and quantitation of *Leishmania donovani* for the diagnosis of Visceral Leishmaniasis patients and the monitoring of their response to treatment. PLoS ONE.

[36] Chowdhury R., Ghosh P., Khan M.A.A., Hossain F., Faisal K., Nath R., Baker J., El Wahed A.A., Nath S.M.P. (2020). Evaluation of Rapid Extraction Methods Coupled with a Recombinase Polymerase Amplification Assay for Point-of-Need Diagnosis of Post-Kala-Azar Dermal Leishmaniasis. Trop. Med. Infect. Dis..

[37] Malhotra I., Dent A., Mungai P., Muchiri E., King C.L. (2005). Real-Time Quantitative PCR for Determining the Burden of *Plasmodium falciparum* Parasites during Pregnancy and Infancy. J. Clin. Microbiol..

[38] Guirou E.A., Schindler T., Hosch S., Donfack O.T., Yoboue C.A., Krähenbühl S., Deal A., Cosi G., Gondwe L., Mwangoka G. (2020). Molecular malaria surveillance using a novel protocol for extraction and analysis of nucleic acids retained on used rapid diagnostic tests. Sci. Rep..

[39] Grignard L., Nolder D., Sepúlveda N., Berhane A., Mihreteab S., Kaaya R., Phelan J., Moser K., van Schalkwyk D.A., Campino S. (2020). A novel multiplex qPCR assay for detection of *Plasmodium falciparum* with histidine-rich protein 2 and 3 (pfhrp2 and pfhrp3) deletions in polyclonal infections. EBioMedicine.

[40] Swan H., Sloan L., Muyombwe A., Chavalitshewinkoon-Petmitr P., Krudsood S., Leowattana W., Wilairatana P., Looareesuwan S., Rosenblatt J. (2005). Evaluation of a real-time polymerase chain reaction assay for the diagnosis of malaria in patients from Thailand. Am. J. Trop. Med. Hyg..

[41] Nuin N.A., Tan A.F., Lew Y.L., Piera K.A., William T., Rajahram G.S., Jelip J., Dony J.F., Mohammad R., Cooper D.J. (2020). Comparative evaluation of two commercial real-time PCR kits (QuantiFast™ and abTES™) for the detection of *Plasmodium knowlesi* and other *Plasmodium* species in Sabah, Malaysia. Malar. J..

[42] Hofmann N., Mwingira F., Shekalaghe S., Robinson L.J., Mueller I., Felger I. (2015). Ultra-sensitive detection of *Plasmodium falciparum* by amplification of multi-copy subtelomeric targets. PLoS Med..

[43] Demas A., Oberstaller J., DeBarry J., Lucchi N.W., Srinivasamoorthy G., Sumari D., Kabanywanyi A.M., Villegas L., Escalante A.A., Kachur S.P. (2011). Applied genomics: Data mining reveals species-specific malaria diagnostic targets more sensitive than 18S rRNA. J. Clin. Microbiol..

[44] Haanshuus C.G., Mohn S.C., Mørch K., Langeland N., Blomberg B., Hanevik K. (2013). A novel, single-amplification PCR targeting mitochondrial genome highly sensitive and specific in diagnosing malaria among returned travellers in Bergen, Norway. Malar. J..

[45] Xu W., Morris U., Aydin-Schmidt B., Msellem M.I., Shakely D., Petzold M., Björkman A., Mårtensson A. (2015). SYBR Green Real-Time PCR-RFLP Assay Targeting the *Plasmodium* Cytochrome B Gene—A Highly Sensitive Molecular Tool for Malaria Parasite Detection and Species Determination. PLoS ONE.

[46] Farrugia C., Cabaret O., Botterel F., Bories C., Foulet F., Costa J.M., Bretagne S. (2011). Cytochrome b Gene Quantitative PCR for Diagnosing *Plasmodium falciparum* Infection in Travelers. J. Clin. Microbiol..

[47] Wang B., Han S.-S., Cho C., Han J.-H., Cheng Y., Lee S.-K., Galappaththy G.N.L., Thimasarn K., Soe M.T., Oo H.W. (2014). Comparison of Microscopy, Nested-PCR, and Real-Time-PCR Assays Using High-Throughput Screening of Pooled Samples for Diagnosis of Malaria in Asymptomatic Carriers from Areas of Endemicity in Myanmar. J. Clin. Microbiol..

[48] Ponchel F., Toomes C., Bransfield K., Leong F.T., Douglas S.H., Field S.L., Bell S.M., Combaret V., Puisieux A., Mighell A.J. (2003). Real-time PCR based on SYBR-Green I fluorescence: An alternative to the TaqMan assay for a relative quantification of gene rearrangements, gene amplifications and micro gene deletions. BMC Biotechnol..

