# Supplementary material for: An Optimized Real-Time qPCR Method for the Effective Detection of Human Malaria Infections
Source: Diagnostics (Basel). 2021 Apr 21;11(5):736. doi: 10.3390/diagnostics11050736 (PMC8142979; doi:10.3390/diagnostics11050736)
Supplement: Supplementary file 1 [file diagnostics-11-00736-s001.zip › Supplementary S1.pdf]

Supplementary (S1): Table 1: Primers with amplicon size and sequences for [1-4] species detection.

Species	Amplicon size	Primers	Sequence of Primer
P. falciparum	206	rFAL1 (Forward)	TTAAACTGGTTTGGGAAAACCAAATATATT
		rFal2 (Reverse)	ACACAATGAACTCAATCATGACTACCCGTC
P. vivax	121	rVIV1 (Forward)	CGCTTCTAGCTTAATCCACATAACTGATAC
		rVIV2 (Reverse)	ACTTCCAAGCCGAAGCAAAGAAAGTCCTTA
P. malariae	145	rMAL1 (Forward)	ATAACATAGTTGTACGTTAAGAATAACCGC
		rMAL2 (Reverse)	AAAATTCCCATGCATAAAAAATTATACAAA
P. ovale	226	rOVA1 (Forward)	ATCTCTTTTGCTATTTTTTAGTATTGGAGA
		rOVA2 (Reverse)	ATCTAAGAATTTCACCTCTGACATCTG
P. knowlesi	153	Pmk8 (Forward)	GTTAGCGAGAGCCACAAAAAAGCGAAT
		Pmkr9 (Reverse)	ACTCAAAGTAACAAAATCTTCCGTA

DNA Extraction protocol

Quick-Start Protocol **September 2018**

QIAamp® Blood Mini Kit

The QIAamp Blood Mini Kit (cat. nos. 51104 and 51106) can be stored at room temperature (15–25°C) for up to 12 months. Reconstituted QIAGEN Protease is stable for 12 months when stored at 2–8°C.

Further information

- QIAamp DNA Mini and Blood Mini Handbook: **www.qiagen.com/HB-0329**
- Safety Data Sheets: **www.qiagen.com/safety**
- Technical assistance: **support.qiagen.com**

Notes before starting

- Perform all centrifugation steps at room temperature (15–25°C).
- Dissolve any precipitates in Buffer AL by warming at 56°C until the precipitate has dissolved.
- Add ethanol to Buffer AW1 and Buffer AW2 concentrates, as indicated on the bottle.
- Add Protease Solvent to lyophilized QIAGEN Protease, as indicated on the label.
- Equilibrate samples to room temperature (15–25°C).
- Preheat a water bath or heating block to 56°C.

Procedure

1. Pipet 20 µl QIAGEN Protease into a 1.5 ml microcentrifuge tube. Add 200 µl sample.

   If the sample volume is less than 200 µl, add the appropriate volume of PBS.

2. Add 200 µl Buffer AL. Mix thoroughly by vortexing.
3. Incubate at 56°C for 10 min. Briefly centrifuge the 1.5 ml microcentrifuge tube to remove drops from the lid.

Sample to Insight

4. Add 200 µl ethanol (96–100%). Mix thoroughly by vortexing. Briefly centrifuge the tube to remove drops from the lid.
5. Pipet the mixture onto the QIAamp Mini spin column (in a 2 ml collection tube) and centrifuge at 6000 x g (8000 rpm) for 1 min. Discard the flow-through and collection tube.

   Note: When preparing DNA from buffy coat or lymphocytes, centrifugation at full speed is recommended to avoid clogging.
6. Place the QIAamp Mini spin column in a new 2 ml collection tube and add 500 µl Buffer AW1. Centrifuge at 6000 x g (8000 rpm) for 1 min. Discard the flow-through and collection tube.
7. Place the QIAamp Mini spin column in a new 2 ml collection tube and add 500 µl Buffer AW2. Centrifuge at full speed (20,000 x g; 14,000 rpm) for 3 min. Discard the flow-through and collection tube.
8. **Recommended**: Place the QIAamp Mini spin column in a new 2 ml collection tube (not provided) and centrifuge at full speed for 1 min. This eliminates the chance of possible Buffer AW2 carryover.
9. Place the QIAamp Mini spin column in a new 1.5 ml microcentrifuge tube (not provided), add 200 µl Buffer AE or distilled water and incubate at room temperature (15–25°C) for 1 min. Centrifuge at 6000 x g (8000 rpm) for 1 min to elute the DNA.

References:

1. Snounou, G. and B. Singh, *Nested PCR Analysis of Plasmodium Parasites*, in *Malaria Methods and Protocols: Methods and Protocols*, D.L. Doolan, Editor. 2002, Humana Press: Totowa, NJ. p. 189-203.
2. Snounou, G., et al., *High sensitivity of detection of human malaria parasites by the use of nested polymerase chain reaction.* Mol Biochem Parasitol, 1993. **61**(2): p. 315-20.
3. Singh, B., et al., *A large focus of naturally acquired Plasmodium knowlesi infections in human beings.* Lancet, 2004. **363**(9414): p. 1017-24.
4. Singh, B., et al., *A genus- and species-specific nested polymerase chain reaction malaria detection assay for epidemiologic studies.* Am J Trop Med Hyg, 1999. **60**(4): p. 687-92.
