# Supplementary material for: An Optimized Real-Time qPCR Method for the Effective Detection of Human Malaria Infections
Source: Diagnostics (Basel). 2021 Apr 21;11(5):736. doi: 10.3390/diagnostics11050736 (PMC8142979; doi:10.3390/diagnostics11050736)

Supplementary S2

Figure 1 (S2): The product from real-time analytical sensitivity PCR has been subjected to gel electrophoresis for further confirmation. The gel products are from high to low concentration from left to right with 100bp DNA ladders on both sides. The 9th and 10th dilution shows the absence of products and thus verify the sensitivity of the *Pf* analytical assay up to 0.064 parasite/µL.

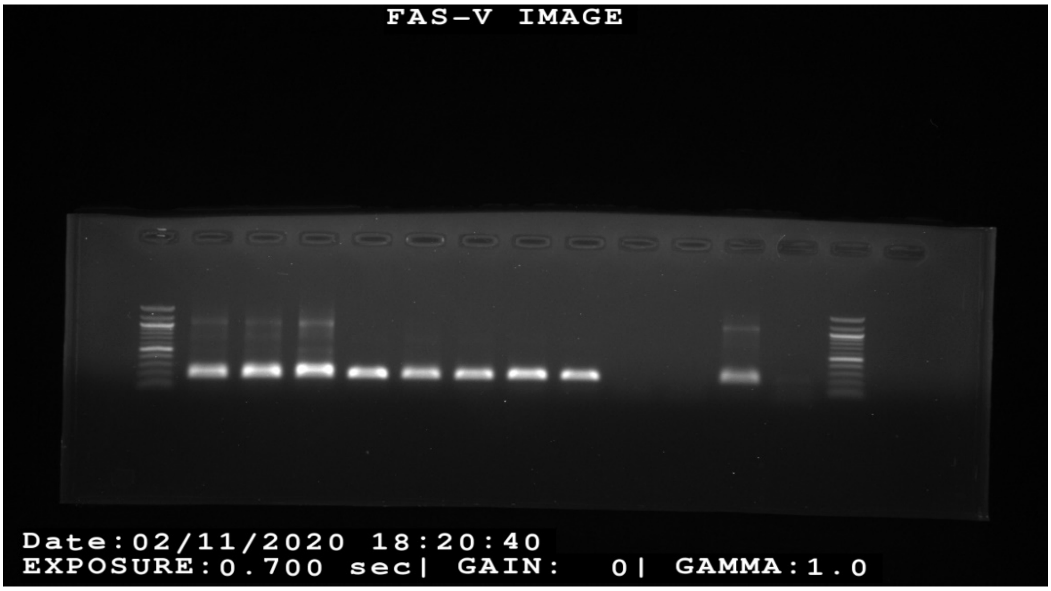

Figure 2 (S2): The product from real-time analytical sensitivity PCR has been subjected to gel electrophoresis for further confirmation. The gel products are from high to low concentration from left to right with 100bp DNA ladders on both sides. The 8th dilution shows the absence of products and thus verify the sensitivity of the *Pv* analytical assay up to 1.6 parasites/µL.

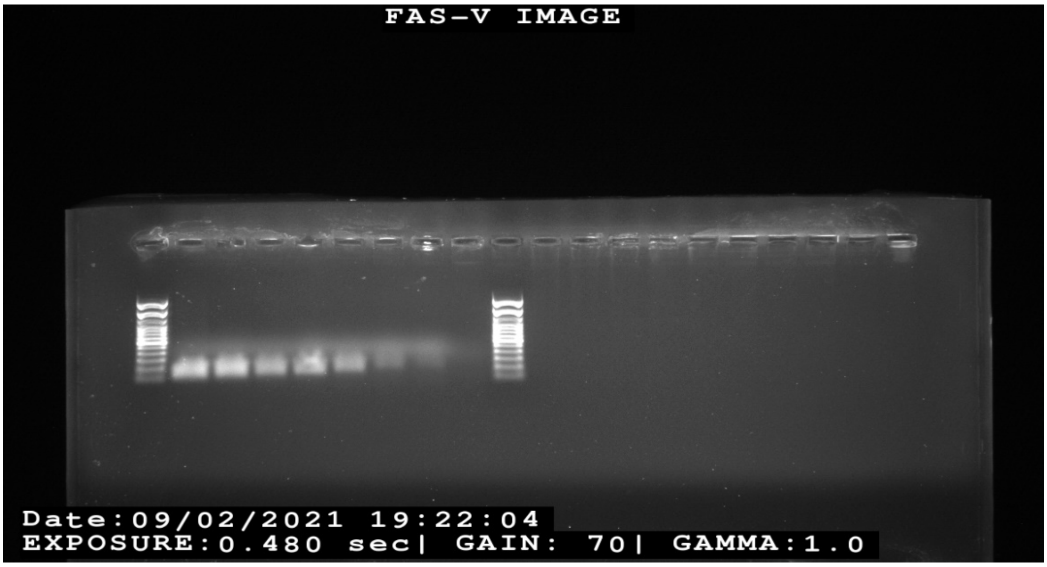

Figure 3 (S2): The product from real-time analytical sensitivity PCR has been subjected to gel electrophoresis for further confirmation. The gel products are from high to low concentration from left to right with 100bp DNA ladders on both sides. The 7th dilution shows the absence of products and thus verify the sensitivity of the *Pm* analytical assay up to 0.32 parasites/µL.

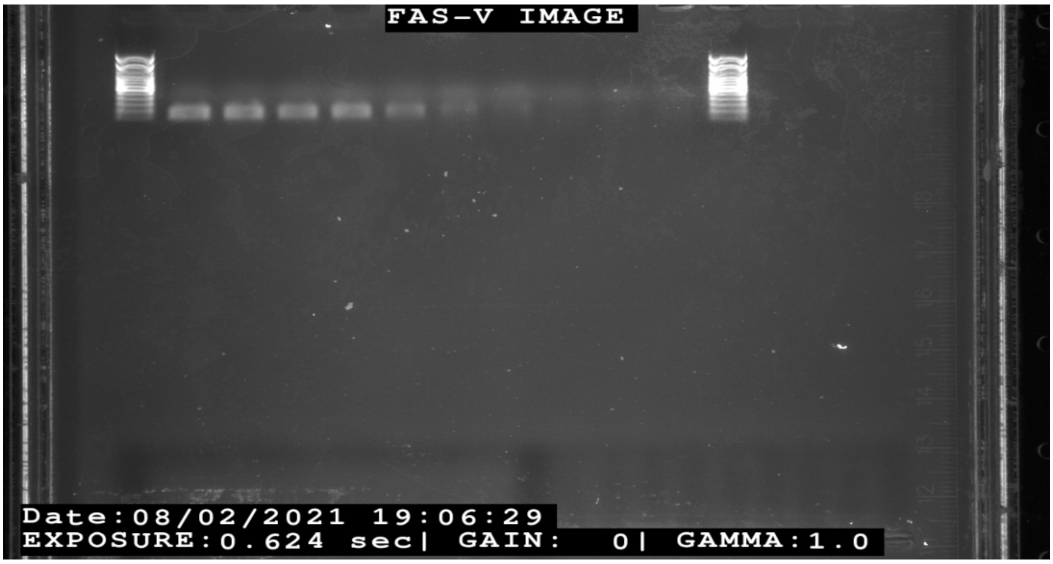

Supplement: Supplementary file 1 [file diagnostics-11-00736-s001.zip › Supplementary S2.pdf]
